# *Lactobacillus brevis* GKJOY Supplementation Ameliorates Oxidative Stress and Reproductive Dysfunction in Male Rats with Polystyrene Microplastics-Induced Reproductive Toxicity

**DOI:** 10.3390/ijms26104533

**Published:** 2025-05-09

**Authors:** Yi-Yuh Hwang, Sabri Sudirman, Yu-Chen Hsu, Chin-Chu Chen, Fanbin Kong, Deng-Fwu Hwang, Zwe-Ling Kong

**Affiliations:** 1 Department of Food Science, National Taiwan Ocean University, Keelung 20224, Taiwan; 2graceyy@gmail.com (Y.-Y.H.); amy10150106@gmail.com (Y.-C.H.); dfhwang@mail.ntou.edu.tw (D.-F.H.); 2 Fisheries Product Technology, Faculty of Agriculture, Universitas Sriwijaya, Indralaya 30662, Indonesia; sabrisudirman@unsri.ac.id; 3 Biotech Research Institute, Grape King Bio Ltd., Taoyuan 32542, Taiwan; gkbioeng@grapeking.com.tw; 4 Department of Food Science and Technology, Collage of Agricultural and Environmental Sciences, University of Georgia, Athens, GA 30602, USA; fkong@uga.edu

**Keywords:** *Lactobacillus brevis* GKJOY, male reproductive, oxidative stress, polystyrene microplastics

## Abstract

The growing demand for plastic products has led to an increase in human exposure to microplastics (MPs). MPs have been shown to have detrimental effects on reproductive function, while probiotics have demonstrated promise in enhancing fertility. This study aimed to determine the protective effects of *Lactobacillus brevis* GKJOY against reproductive damage induced by polystyrene microplastics (PS-MPs) in male rats. In the cell study, LC540 cells were treated with *L. brevis* GKJOY postbiotic (PGK), gamma-aminobutyric acid (GABA), and PS-MPs to evaluate their effects on cell viability and reactive oxygen species (ROS) production. In the animal experiment, rats were treated with a low dose of *L. brevis* GKJOY (GK1X, 50 mg/kg), a medium dose (GK2X, 100 mg/kg), or a high dose (GK4X, 200 mg/kg). The results showed that PGK and GABA reduced the levels of ROS and protected against oxidative stress. In contrast, PS-MPs increased ROS levels and had harmful effects on cell viability. In the animal study, testicular injuries caused by PS-MPs led to disruption of the hypothalamic–pituitary–gonadal (HPG) axis and a decrease in reproductive hormone levels. However, treatment with *L. brevis* GKJOY reduced oxidative stress and pro-inflammatory cytokine levels, restored hormonal imbalances, and led to significant improvements. *L. brevis* GKJOY effectively mitigated reproductive damage in male rats due to its dual function as a probiotic and neurotransmitter modulator. In conclusion, *L. brevis* GKJOY, which functions as both a probiotic and a GABA producer, may offer superior protection against male reproductive damage.

## 1. Introduction

Microplastics (MPs) are tiny plastic granules used as scrubbers in cosmetics and air-blasting, as well as small plastic fragments ranging from 1 μm to 5 mm in size, derived from the breakdown of macroplastics [1,2]. In recent years, environmental concerns regarding MPs have been increasing. The small size of MPs makes them easily ingested by marine organisms, causing bioaccumulation when their uptake from water, sediment, or prey exceeds their excretion capacity [3]. Among the various plastic types, polystyrene microplastics (PS-MPs) are among the most prevalent, known for their extensive application in packaging, consumer products, and medical devices [4]. PS-MPs are widely utilized as a plastic material, making them a frequently used model particle for investigating biological consequences. The potential of PS-MPs to carry chemicals and cross biological barriers raises concerns regarding their impact on human health [5,6].

Numerous studies have shown that PS-MPs can lead to liver lipid metabolism disorders, microbiota dysbiosis, and gut inflammation in marine organisms [7,8]. A previous study demonstrated that small-sized MPs are particularly hazardous due to their higher bioavailability and longer residence time in the body [9]. Other studies also show that PS-MPs induce reproductive toxicity in mice through oxidative stress and activation of the p38 MAPK signaling pathway, causing oxidative damage to testicular tissue and apoptosis of sperm cells, ultimately leading to infertility [10,11]. Additionally, the detrimental effects of PS-MPs on health include oxidative stress, disruption of the blood–testis barrier, and impacts on sperm quality [12,13]. Animal studies have shown that PS-MPs induce the production of pro-inflammatory cytokines such as tumor necrosis factor (TNF)-α, interleukins (IL)-6, and IL-1β, leading to abnormalities in sperm quality in mice [1,14]. These findings highlight the critical need to understand and address the impact of PS-MPs on the reproductive system to safeguard both human and animal fertility, as well as to explore potential treatments to mitigate the harmful effects of PS-MPs when they are taken up by the body.

Probiotics are defined as bacteria that promote health when administered to a host in adequate amounts. These bacteria are typically regarded harmless because of their ability to survive in the body and assist in curing and preventing diseases. Numerous clinical trials have demonstrated that probiotic strains are both safe and effective in delivering health benefits to users [15,16]. Previous studies have reported that probiotics and prebiotics improve sperm quality in patients with asthenospermia [17,18]. Animal studies have shown that probiotics enhance sperm motility in zebrafish and rats by balancing hormones, reducing oxidative stress, and decreasing inflammation [19,20].

*Lactobacillus* and *Bifidobacterium* are the two most commonly used genera when it comes to probiotics [21]. *Lactobacillus* is a genus of Gram-positive, rod-shaped bacteria and is one of the most abundant and diverse groups of bacteria in the human body [22]. One such beneficial bacterium is *Lactobacillus brevis*, known for its dual benefits as both a probiotic and a gamma-aminobutyric acid (GABA) producer [23,24]. Various *L. brevis* strains have been reported for their beneficial effects. Animal studies have shown that GABA-producing *L. brevis* FPA3709 has an antidepressant effect [25]. Additionally, supplementation with specific *L. brevis* strains (SAD and BIOTECH 1766) mitigated oxidative stress [26,27]. A previous study also reported that *L. brevis* IBRC-M10790 reduced pro-inflammatory cytokine levels in an in vitro inflammatory bowel disease model [28]. However, there is a lack of research on the effects of *L. brevis* on male reproduction in rat models, especially *L. brevis* GKJOY. We hypothesized that *L. brevis* GKJOY would have beneficial effects on the male reproductive system. Therefore, this study aimed to investigate the effects of *L. brevis* GKJOY on reproductive system damage induced by polystyrene microplastics.

## 2. Results

### 2.1. Leydig Cell (LC540) Viability

**Figure 1:**
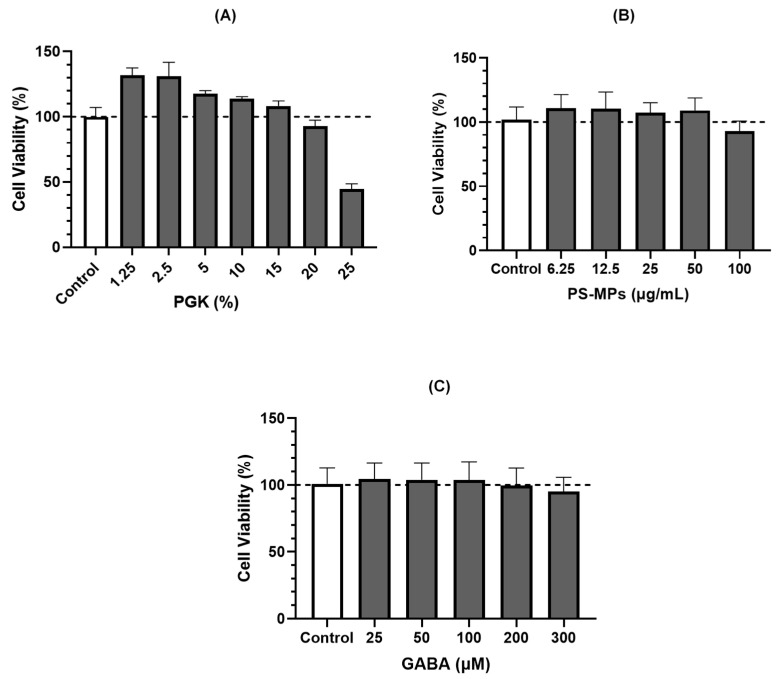
Cell viability of LC540 was treated with (**A**) PGK, (**B**) PS-MPs, and (**C**) GABA for 24 h. The results are presented as mean ± SD (*n* = 8).

The viability of Leydig cells (LC540) treated with different concentrations of various substances (PGK, PS-MPs, and GABA) is shown in Figure 1. LC540 cells were co-cultured with different PGK concentrations for 24 h. The results indicated that the cell survival rate dropped below 100% when the PGK concentration exceeded 15%. This demonstrates the cytotoxicity of PGK against LC540 cells. Thus, 15% PGK was selected for subsequent experiments (Figure 1A). LC540 cells were co-cultured with PS-MPs at different concentrations for 24 h. It was observed that concentrations of PS-MPs greater than 50 μg/mL caused the cell survival rate to drop below 100%. Therefore, 50 μg/mL PS-MPs was chosen for further experiments (Figure 1B). Next, the effect of different GABA concentrations on LC540 cell survival was measured after a 24 h co-culture. The results showed that GABA concentrations above 200 μM caused the cell survival rate to decrease below 100%. Therefore, 200 μM GABA was selected for the subsequent experiments (Figure 1C).

### 2.2. Reactive Oxygen Species Production in LC540 Cells

**Figure 2:**
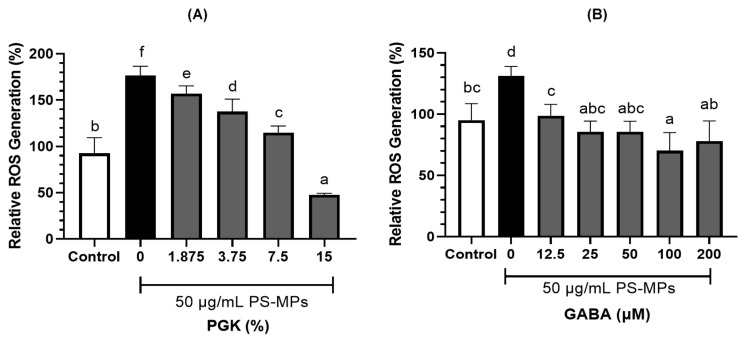
Effects of (**A**) PGK and (**B**) GABA on PS-MPs induced relative ROS generation in LC540 cells. The results are presented as mean ± SD (*n* = 8). Different letters (a–f) above bars represent significant difference (*p* < 0.05) as analyzed by the Tukey test.

The effects of PGK and GABA on reactive oxygen species (ROS) production in LC540 cells treated with PS-MPs are shown in Figure 2. The ROS levels were significantly (*p* < 0.05) higher in the untreated PS-MPs group. Conversely, ROS production decreased in a concentration-dependent manner upon co-culture with various concentrations of PGK (Figure 2A). Similarly, a significant (*p* < 0.05) decrease in ROS production was observed when cells were co-cultured with GABA (Figure 2B).

### 2.3. Rats’ Body Weight and Organ Weight

**Figure 3:**
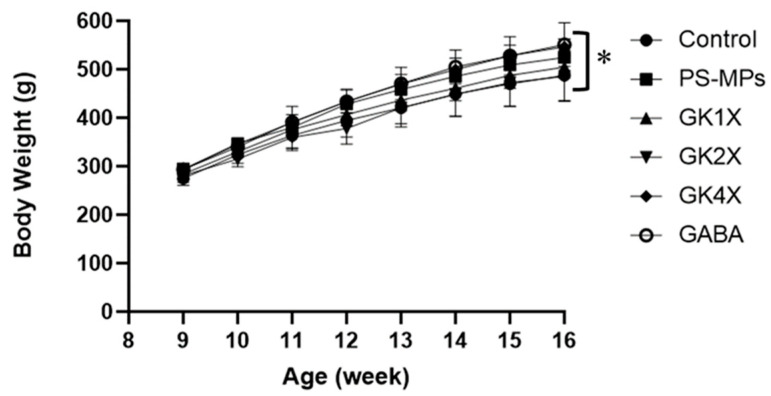
Average body weight of rats during the treatments. The data are expressed as mean ± SD (*n* = 6). *, represent no significance (*p* > 0.05) in each group. GABA, γ-aminobutyric acid; GK, *L. brevis* GKJOY; PS-MPs, polystyrene microplastics.

The body and organ weights of the rats after eight weeks of treatment are shown in Figure 3 and Table 1, respectively. The results indicated that there were no statistically significant (*p* < 0.05) differences in body weight and organ (liver, spleen, kidneys, abdominal fat, testes, and their associated epididymal fat pads) weight alterations among the various groups. This suggests that the administration of different doses of *L. brevis* GKJOY and GABA did not affect animal body weight or organ weight.

### 2.4. Malondialdehyde and Nitric Oxide Concentrations

**Figure 4:**
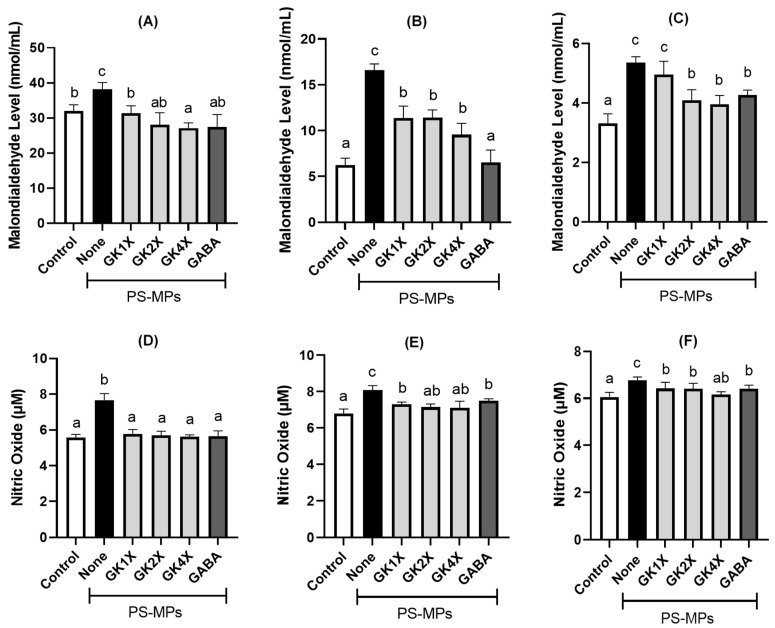
Effects of *Lactobacillus brevis* GKJOY and GABA on the malondialdehyde (MDA) concentrations in (**A**) plasma, (**B**) testis, and (**C**) sperm of rats and nitric oxide (NO) concentrations in (**D**) plasma, (**E**) testis, and (**F**) sperm of rats after 8 weeks of treatments. Data are expressed as mean ± SD (*n* = 6). Different letters (a–c) above bars represent significant different (*p* < 0.05) as analyzed by the Tukey test. GABA, γ-aminobutyric acid; GK, *L. brevis* GKJOY; PS-MPs, polystyrene microplastics.

The effects of *L. brevis* GKJOY and GABA treatments on malondialdehyde (MDA) and nitric oxide (NO) levels are shown in Figure 4. A significant (*p* < 0.05) increase in MDA levels in the plasma, testis, and sperm of the rats was observed in the untreated PS-MPs group compared to that in the control group (Figure 4A–C). However, a significant (*p* < 0.05) reduction in MDA levels was observed in the groups treated with various doses of *L. brevis* GKJOY and GABA compared with the untreated PS-MPs group. Additionally, NO levels in the plasma, testis, and sperm of the rats were significantly (*p* < 0.05) higher in the untreated PS-MPs group than in the control group (Figure 4D–F). Similar to the MDA levels, the levels of NO were also significantly (*p* < 0.05) decreased in the groups treated with various doses of *L. brevis* GKJOY and GABA compared to the untreated PS-MPs group after 8 weeks of treatment. These results indicate that *L. brevis* GKJOY and GABA can attenuate PS-MP-induced oxidative stress in rats.

### 2.5. Glutathione Peroxidase Activity

**Figure 5:**
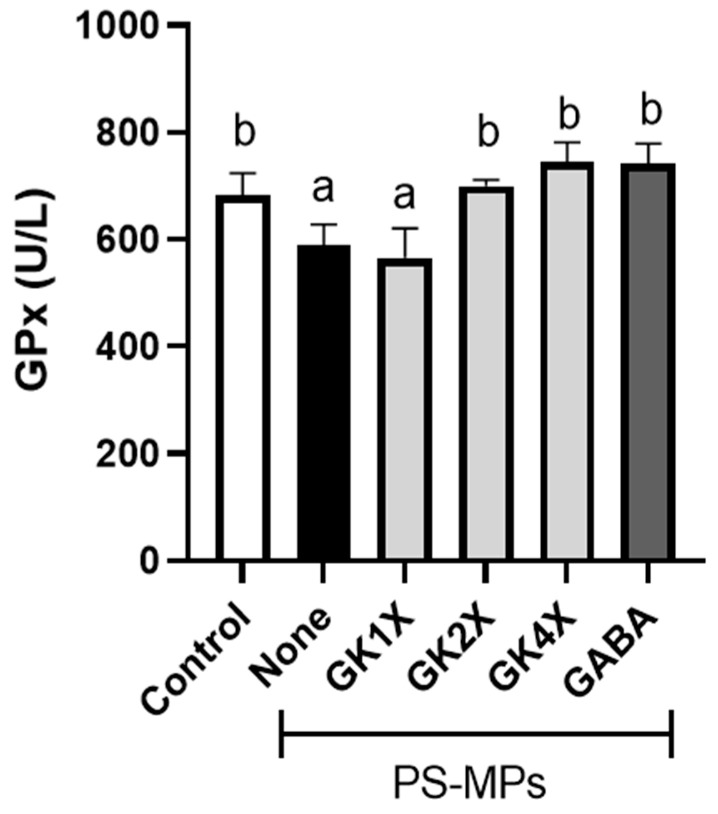
Effects of *Lactobacillus brevis* GKJOY and GABA on glutathione peroxidase (GPx) activity in plasma of rats after 8 weeks of treatments. Data are expressed as mean ± SD (*n* = 6). Different letters (a, b) above bars represent significant difference (*p* < 0.05) as analyzed by the Tukey test. GABA, γ-aminobutyric acid; GK, *L. brevis* GKJOY; PS-MPs, polystyrene microplastics.

The enzymatic antioxidant activity of plasma in rats after eight weeks of treatment, such as glutathione peroxidase (GPx), is shown in Figure 5. PS-MPs exposure significantly (*p* < 0.05) reduced glutathione peroxidase (GPx) activity in the untreated PS-MPs group. However, supplementation with *L. brevis* GKJOY and GABA for eight weeks significantly (*p* < 0.05) enhanced GPx activity.

### 2.6. Pro-Inflammatory Cytokine Expressions

**Figure 6:**
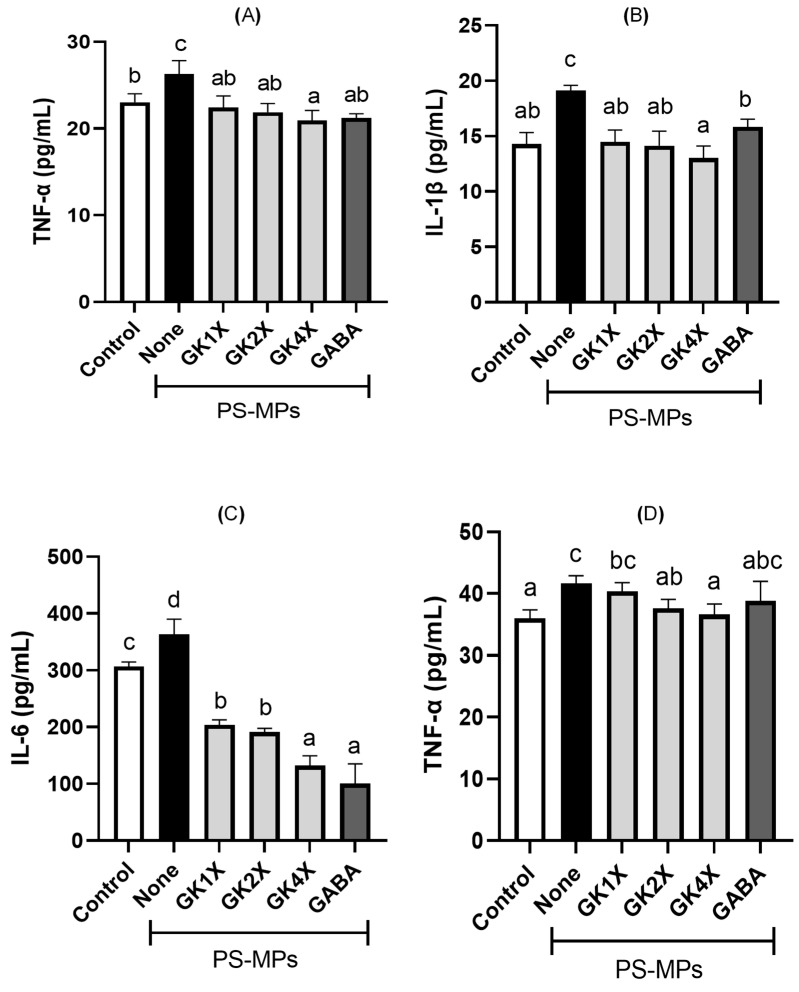
Effects of *Lactobacillus brevis* GKJOY and GABA on the (**A**) tumor necrosis factor-α (TNF-α), (**B**) interleukin (IL)-1β (**C**) IL-6 expressions in plasma of rats, and (**D**) TNF-α expression in testes of rats after 8 weeks of treatments. Data are expressed as mean ± SD (*n* = 6). Different letters (a–d) above bars represent significant difference (*p* < 0.05) as analyzed by the Tukey test. GABA, γ-aminobutyric acid; GK, *L. brevis* GKJOY; PS-MPs, polystyrene microplastics.

Pro-inflammatory expression in the rat plasma and testes is shown in Figure 6. A significant increase (*p* < 0.05) in TNF-α, IL-1β, and IL-6 in rat plasma (Figure 6A–C) and TNF-α in rat testes (Figure 6D) was observed in the untreated PS-MPs group compared to the control group. These expressions were significantly (*p* < 0.05) reduced after eight weeks of treatment with various doses of *L. brevis* GKJOY, both in the plasma and testes of the rats, especially in medium (GK2X) and high doses (GK4X), compared to the untreated PS-MPs group. GAMA treatment also significantly (*p* < 0.05) reduced the levels of these pro-inflammatory cytokines in the rat plasma. These results demonstrate that *L. brevis* GKJOY can attenuate PS-MP-induced inflammation in the rat plasma and testes.

### 2.7. Kisspeptin and Reproductive Hormone Levels

**Figure 7:**
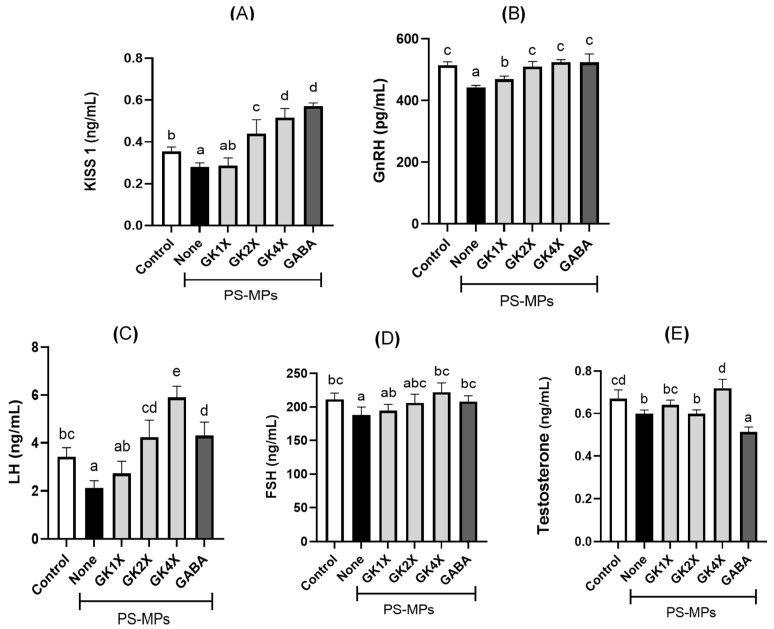
Effects of *Lactobacillus brevis* GKJOY and GABA on the (**A**) kisspeptin, (**B**) gonadotropin-releasing hormone (GnRH), (**C**) luteinizing hormone (LH), (**D**) follicle stimulating hormone (FSH), and (**E**) testosterone levels in plasma of rats after 8 weeks of treatments. Data are expressed as mean ± SD (*n* = 6). Different letters (a–e) above bars represent significant difference (*p* < 0.05) as analyzed by the Tukey test. GABA, γ-aminobutyric acid; GK, *L. brevis* GKJOY; PS-MPs, polystyrene microplastics.

The levels of kisspeptin and reproductive hormones in the plasma of rats after eight weeks of treatment with *L. brevis* GKJOY and GABA are shown in Figure 7. A significant (*p* < 0.05) reduction in kisspeptin levels was observed in the untreated PS-MPs group compared with that in the control group (Figure 7A). Similarly, several reproductive hormones in rat plasma, such as gonadotropin-releasing hormone (GnRH), luteinizing hormone (LH), follicle-stimulating hormone (FSH), and testosterone, were also significantly (*p* < 0.05) decreased in the untreated PS-MPs group compared to the control group (Figure 7B–E). However, the levels of these hormones (GnRH and LH) improved following the subsequent administration of medium and high doses of *L. brevis* GKJOY (GK2X and GK4X, respectively) and GABA for 8 weeks. FSH and testosterone levels also increased with a high dose of *L. brevis* GKJOY (GK4X). These findings indicate that treatment with *L. brevis* GKJOY and GABA ameliorated PS-MP-induced perturbations in reproductive hormone homeostasis, thereby fostering the maintenance of reproductive hormone equilibrium.

### 2.8. 5-Hydroxytryptamine and 5-Hydroxytryptamine Receptor 1A Expression

**Figure 8:**
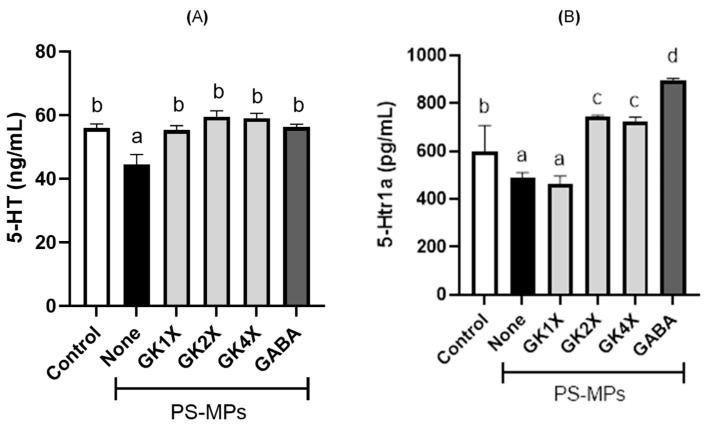
Effects of *Lactobacillus brevis* GKJOY and GABA on the (**A**) 5-hydroxytryptamine (5-HT) and (**B**) 5-hydroxytryptamine receptor 1A (5-Htr1a) expressions in rat brain homogenates after 8 weeks of treatments. Data are expressed as mean ± SD (*n* = 6). Different letters (a–d) above bars represent significant difference (*p* < 0.05) as analyzed by the Tukey test. GABA, γ-aminobutyric acid; GK, *L. brevis* GKJOY; PS-MPs, polystyrene microplastics.

The expression levels of serotonin (5-hydroxytryptamine) and its receptor (5-hydroxytryptamine receptor 1A) in the rat brain homogenates are shown in Figure 8. A significant (*p* < 0.05) reduction in serotonin and serotonin receptor levels was observed in the untreated PS-MPs group compared with the control group. However, the level of serotonin significantly (*p* < 0.05) improved after treatment with different doses of *L. brevis* GKJOY and GABA (Figure 8A). The serotonin receptor level also significantly (*p* < 0.05) increased after treatment with medium and high doses of *L. brevis* GKJOY and GABA, respectively (Figure 8B).

### 2.9. Testis Histopathology

**Figure 9:**
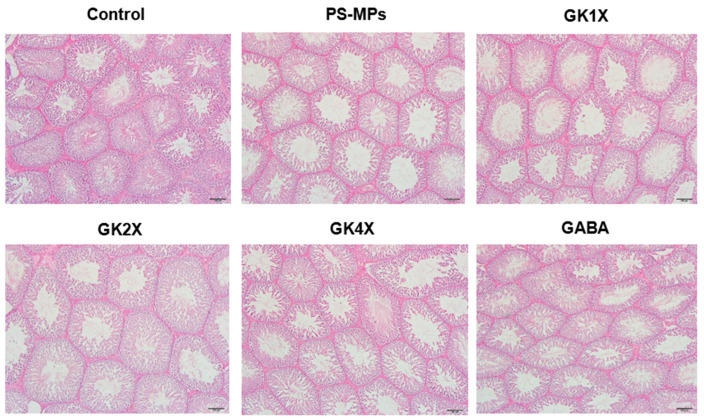
Effects of *Lactobacillus brevis* GKJOY and GABA on seminiferous tubules of the testis in rats after 8 weeks of treatment. Representative images of hematoxylin and eosin (H&E) sections in the testis of each group. The scale bar indicates 100 µm. GABA, γ-aminobutyric acid; GK, *L. brevis* GKJOY; PS-MPs, polystyrene microplastics.

The testicular histopathology of the rats is shown in Figure 9, and the thickness of the epithelium and the area of the seminiferous lumen tubules in the testes of rats are shown in Table 2. Normal morphology of seminiferous tubules with closely arranged and complete spermatocytes was observed in the control group (Figure 9). However, the untreated PS-MPs group showed a loss of epithelial cells, thinning of the tubule wall, enlargement of the lumen area, and severe vacuolation (Figure 9). In contrast, the irregularity of epithelial cells was reduced, and germ cells were arranged normally after treatment with medium and high doses of *L. brevis* GKJOY (GK2X and GK4X, respectively) and GABA (Figure 9).

A significant (*p* < 0.05) decrease in the epithelial thickness of rat testes was observed in the untreated PS-MPs group compared to that in the control group (Table 2). Consequently, there was an increase in seminiferous tubule area in this group. However, the thickness of the epithelium significantly (*p* < 0.05) improved after treatment with medium and high doses of *L. brevis* GKJOY (GK2X and GK4X, respectively) and GABA compared to the untreated PS-MPs group. Additionally, a significant (*p* < 0.05) improvement in the seminiferous tubule area was observed after treatment with various doses of *L. brevis* GKJOY and GABA compared with the untreated PS-MPs group.

## 3. Discussion

This study successfully demonstrated the beneficial effects of *Lactobacillus brevis* GKJOY postbiotics (PGK) on Leydig cells (LC540) and *L. brevis* GKJOY supplementation on PS-MP-induced reproductive toxicity in male rats. Figure 1 shows that LC540 cell survival rate decreased below 100% when the PGK concentration exceeded 15%, GABA concentrations above 200 μM, and PS-MPs greater than 50 μg/mL. These data indicated that the maximum concentration of these substances was selected for subsequent experiments, such as the evaluation of reactive oxygen species (ROS) production in LC540 cells. Figure 2 shows the increase in ROS production in LC540 cells in the untreated PS-MPs group. A previous study also reported increased ROS production in LC540 cells with exposure to PS-MPs [29]. Additionally, elevated ROS production was observed in cells induced by PS- MPs [30]. Treatment using PGK postbiotic successfully reduced ROS levels in a concentration-dependent manner. This indicated that the postbiotic PGK exhibited antioxidant properties against PS-MPs exposure in LC540 cells. A previous study reported that *Levilactobacillus brevis* BK3 postbiotics also reduced intracellular ROS levels during H_2_O_2_-induced oxidative damage [31].

In the animal study, the results indicated that different doses of *L. brevis* GKJOY did not affect the rats’ body weight or organ weight, including the liver, spleen, kidneys, abdominal fat, and testes, or their associated epididymal fat pads (Figure 3 and Table 1). A previous study also reported that untreated PS-MPs exposure and treatments had no effect on the body weight and organ weight of the rats [29]. On the other hand, PS-MPs exposure elevated the levels of malondialdehyde (MDA) and nitric oxide (NO) in the untreated group (Figure 4). Previous studies have also reported an increase in MDA and NO levels in response to PS-MP/NP exposure [31,32]. This is due to a decrease in antioxidant activity, such as glutathione peroxidase (GPx), as shown in Figure 5. A previous study also reported that PS-MPs exposure reduced antioxidant activities, especially those of glutathione peroxidase, superoxide dismutase, and catalase [29]. MDA and NO are recognized as markers of oxidative stress. MDA is also known as a lipid peroxidation product, whereas NO is a reactive species that can also act as a signaling molecule [33]. An increase in oxidative stress levels can induce impairment of reproductive function [34]. However, treatment with *L. brevis* GKJOY for 8 weeks successfully enhanced GPx activity, accompanied by a reduction in MDA and NO levels. This indicates that *L. brevis* GKJOY exhibited antioxidant activity in rats exposed to PS-MPs. We hypothesized that *L. brevis* GKJOY also increased the total antioxidant capacity, as indicated by a reduction in MDA and NO levels. A previous study also reported that *L. brevis* SAD enhanced the superoxide dismutase (SOD) and catalase levels [26]. Additionally, *Lactobacillus* species also reduce oxidative stress to prevent disease [35].

Some pro-inflammatory cytokines, such as tumor necrosis factor (TNF)-α, interleukin (IL)-1β, and IL-6, were also increased during PS-MPs exposure in the untreated PS-MPs group (Figure 6). A previous study also reported that PS-MPs increase pro-inflammatory cytokines such as TNF-α, IL-1β, and IL-6 in rats via the TLR4/NF-κB/COX-2 pathway [36]. The increase in pro-inflammatory cytokines also has adverse effects on sperm quality, leading to infertility [37]. The reduction in pro-inflammatory cytokine levels was observed in groups treated with *L. brevis* GKJOY. This indicates that *L. brevis* GKJOY exhibits anti-inflammatory properties by attenuating the inflammation induced by PS-MPs. A previous study also reported that probiotic *L. casei* Zhang supplementation decreased pro-inflammatory cytokine production in a rat model [38].

This study also provides compelling evidence that PS-MPs induce significant alterations in reproductive hormone regulation, as evidenced by the marked reduction in kisspeptin protein expression in the plasma (Figure 7A). Kisspeptin, a critical regulator of the hypothalamic–pituitary–gonadal (HPG) axis, plays a vital role in modulating reproductive hormones [39]. The observed reduction in kisspeptin expression in the untreated PS-MPs group suggests a disruption in the HPG axis, potentially leading to impaired reproductive function. Furthermore, the assessment of reproductive hormone concentrations demonstrated a decline in gonadotropin-releasing hormone (GnRH), follicle-stimulating hormone (FSH), luteinizing hormone (LH), and testosterone levels in the untreated PS-MPs group (Figure 7B–D). However, the administration of *L. brevis* GKJOY demonstrated a substantial restorative effect on kisspeptin levels. These changes are accompanied by an increase in reproductive hormone levels. The mechanistic basis for these observations may be the anti-inflammatory and antioxidant properties of *L. brevis*. This highlights the potential of *L. brevis* GKJOY as a therapeutic agent to manage PS-MP-induced reproductive toxicity. Previous studies have reported the protective effects of *L. brevis* against ethanol-induced liver injury, which is mediated through oxidative stress pathways [40,41]. Additionally, probiotics have been shown to enhance sperm motility in zebrafish and rat models by managing hormonal balance and reducing oxidative stress and inflammation [20,41]. Moreover, the combination of *L. paracasei* and oligo-fructosaccharides has been shown to significantly enhanced the production of LH, FSH, and testosterone in patients with idiopathic oligospermia [17].

Alterations in serotonin (5-hydroxytryptamine) concentration and serotonin receptor (5-hydroxytryptamine receptor 1A) expression were observed in the brains of rats exposed to PS-MPs. PS-MPs decreased serotonin concentration and serotonin receptor expression (Figure 8). This condition potentially affects mood, gastrointestinal motility, and immune function [42]. However, treatment with *L. brevis* GKJOY restored serotonin levels and receptor expression, indicating its beneficial effects on neurological and digestive health. Serotonin is known as a neurotransmitter that is intricately involved in male reproductive physiology [43]. A previous study reported that changes in serotonin levels and serotonin transporter expression have notable effects on male sexual behavior and reproductive health [44].

Histological analysis of the seminiferous tubules revealed significant damage induced by PS-MPs, including loss of epithelial cells, thinning of the tubule wall, enlargement of the lumen area, and severe vacuolation (Figure 9). Damage leads to disordered spermatogenic cells and testicular inflammation. Previous studies have reported that PS-MPs impair blood–testis barrier integrity via the p38 MAPK-Nrf2 pathway triggered by oxidative stress [10,45]. However, treatment with *L. brevis* GKJOY mitigated these pathological changes, indicating its protective effects against PS-MP-induced reproductive damage. This may be because of the antioxidative and anti-inflammatory properties of *L. brevis* GKJOY. However, further research is still needed, such as evaluating the effect of *L. brevis* GKJOY on apoptosis-related genes in rat sperm.

Overall, *L. brevis* GKJOY ameliorated the PS-MP-induced reproductive dysfunction. Previous studies have reported that *L. brevis* is known for its dual benefits as a probiotic and gamma-aminobutyric acid (GABA) producer [23,24]. This process involves the activation of the glutamate decarboxylase (GAD) operon, which converts glutamate into GABA. The present study reported that GABA supplementation in PS-MP-induced rats also successfully reduced oxidative stress and inflammation, enhancing reproductive hormone and serotonin levels, which led to improved reproductive health. A previous study also reported reduced oxidative stress and inflammation in an animal model, leading to increased sperm quality [46,47].

## 4. Materials and Methods

### 4.1. Materials

The polystyrene microplastic particles (PS-MPs) were acquired from the Cell-Bio Biotechnology Co., Ltd. (New Taipei City, Taiwan) with a particle size ranging from 0.4 to 0.6 μm and a concentration of 10% (*w*/*v*) (Cat. No. DNM-P004). *Lactobacillus brevis* GKJOY powder was obtained from Grape King Biotechnology Co. Ltd. (Taoyuan, Taiwan). Male Sprague-Dawley (SD) rats were purchased from BioLASCO Taiwan Co. Ltd. (Yilan, Taiwan). The standard dietary feed for the experimental animals was the Lab Diet 5001 Rodent Diet (PMI Nutrition International Inc., St. Paul, MN, USA). Dulbecco’s modified Eagle’s medium (DMEM) F-12 was purchased from Thermo Fisher Scientific (Waltham, MA, USA). 3-[4,5-dimethylthiazol-2-yl]-2,5 diphenyl tetrazolium bromide (MTT), nitrotetrazolium blue chloride (NBT), and phosphate-buffered saline (PBS) were purchased from Sigma-Aldrich (St. Louis, MO, USA). ELISA kits for TNF-α (Cat. No. ab100785), IL-6 (Cat. No. ab100772), and IL-1β (Cat. No. ab100768) were purchased from Abcam Ltd. (Cambridge, MA, USA). ELISA kits for kisspeptin (Cat. No. MBS3809627), gonadotropin-releasing hormone (GnRH, Cat. No. MBS762089), testosterone (Cat. No. MBS9424769), follicle-stimulating hormone (FSH, Cat. No. MBS2021901), and luteinizing hormone (LH, Cat. No. MBS764675) were purchased from MyBioSource (San Diego, CA, USA).

### 4.2. Polystyrene Microplastics (PS-MPs) Solution Preparation

Polystyrene Microplastics (PS-MPs) solutions were prepared at various concentrations according to the required concentration. Briefly, a 10% (*w*/*v*) PS-MP solution was prepared in deionized water and subjected to ultrasonic vibration for 30 min to ensure uniform dispersion of the plastic particles. Fresh suspensions were prepared daily to maintain consistency and to prevent aggregation [48].

### 4.3. Preparation of γ-Aminobutyric Acid Solution

In the cell experiments, *γ*-aminobutyric acid (GABA) powder was dissolved in DMEM-F12 at dosages designed based on the methodology described in a previous study [49]. For animal experiments, a dosage of 17 mg/kg was selected based on the dosage used in a previous study [41].

### 4.4. Cell-Free Supernatant Postbiotic Preparation

The postbiotic metabolites of *L. brevis* GKJOY fermentation (PGK) were prepared according to a previous study [50]. Briefly, *L. brevis* GKJOY [1% (*v*/*v*)] was grown in MRS broth for 24 h at 37 °C. Cells were separated by centrifugation at 10,000× *g* for 10 min at 4 °C. The supernatant was collected as the cell-free supernatant (CFS). The pH of each CFS sample was adjusted to physiological pH (pH 7.2–7.4) using 5 M sodium hydroxide. The CFS containing PKG and all tested PGK were filtered through a 0.22 μm polyethersulfone membrane syringe filter (Millipore, Burlington, MA, USA) prior to cytotoxicity assays.

### 4.5. Leydig Cell Line (LC540) Study

#### 4.5.1. Cell Viability Assay

The Leydig cell line (LC540) was plated in 96-well plates at a density of 6 × 10^5^ cells per well for the cell viability assay, following a previous method [51]. The cells were then exposed to varying concentrations of PS-MPs, PGK, and GABA for 24 h to assess their cytotoxic effects. After the incubation period, the culture medium was removed and 100 μL of MTT reagent (5 mg/mL) was added to each well. Following another incubation period, the formazan crystals formed by viable cells were dissolved in DMSO and the absorbance was measured at 570 nm using a microplate reader. The absorbance values were used as indicators of cell viability and metabolic activity.

#### 4.5.2. Reactive Oxygen Species Assay

The level of reactive oxygen species (ROS) produced in LC540 cells was determined using the fluorescent dye 2,7-dichlorodihydrofluorescein diacetate (H₂DCF-DA) [52]. LC540 cells were plated at a density of 6 × 10 cells per well in 96-well plates. The cells were exposed to varying concentrations of PS-MPs with PGK or PS-MPs with GABA for 24 h. After the incubation period, the culture medium was removed and 100 μL of 10 μM H₂DCFDA solution was added to each well, followed by a 30-min incubation. The fluorescence emitted by dichlorofluorescein (DCF) was measured spectrophotometrically at the excitation and emission wavelengths of 488 and 515 nm, respectively.

### 4.6. Animal Study

#### 4.6.1. Animal Treatment

**Figure 10:**
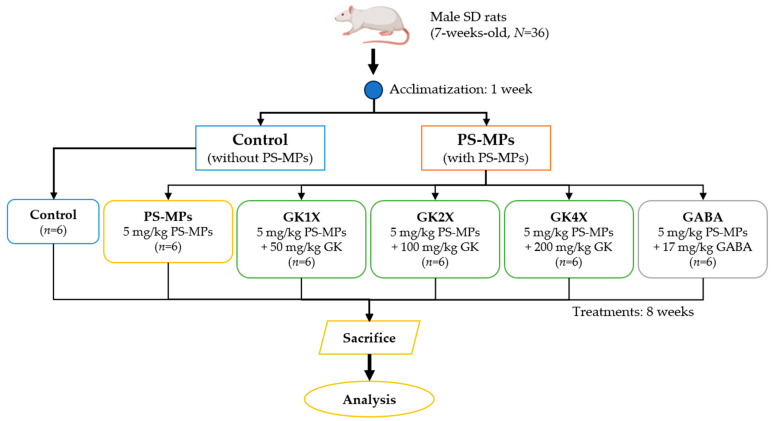
The flowchart of polystyrene microplastics (PS-MPs)-induced reproductive toxicity and *L. brevis* GKJOY (GK) and γ-aminobutyric acid (GABA) treatment for 8 weeks.

Thirty-six healthy adult male (240 ± 10 g) Sprague-Dawley (SD) rats (*N* = 36, 7 weeks-old) were housed under standard laboratory conditions (light/dark cycles of 12 h/12 h, humidity of 40–60%, and a constant temperature of 20 ± 2 °C) in a stainless steel cage and fed standard chow diets. Food and water were provided ad libitum. The animal study protocol was approved by the Institutional Animal Care and Use Committee (IACUC) of National Taiwan Ocean University (IACUC Approval No. 112026 and 17 October 2023). Briefly, the rats were acclimatized for one week and then randomly divided into six groups, with each group consisting of six rats (Figure 10): control group (no treatment), PS-MPs group (fed only PS-MPs Solution, 5 mg/kg), and the *Lactobacillus brevis* GKJOY (GK) treated groups GK1X (5 mg/kg PS-MPs + 50 mg/kg GK), GK2X group (5 mg/kg PS-MPs + 100 mg/kg GK), and GK4X group (5 mg/kg PS-MPs + 200 mg/kg GK). The GABA group was used as a positive control (5 mg/kg PS-MPs + 17 mg/kg GABA). Animal body weights were measured once per week throughout the experiment. All groups except the Control group were administered 5 mg/kg PS-MPs per day by oral gavage for 8 weeks to induce reproductive injury [29]. The *L. brevis* GKJOY doses were chosen according to previous studies [23,41]. All animals were sacrificed at 16 weeks old (8 weeks of treatment). After treatment, rats were fasted for 12 h before sacrifice. Rats were sacrificed in an empty chamber by exposure to CO_2_ until euthanasia according to the previous studies [53,54]. The blood and testes were collected on the day of sacrifice.

#### 4.6.2. Blood Sample Collection

The blood of rats was collected from their abdominal aorta on the day of sacrifice using a heparinized syringe [55]. The blood was then centrifuged at 3000× *g* at 4 °C for 20 min to collect plasma (supernatant). Plasma was stored at −80 °C for further analysis [56].

#### 4.6.3. Tissue Homogenized Preparation

The brain and one testis from each rat were stored at −80 °C. The brain and testis tissues were sliced, washed in PBS, and homogenized in PBS using an automatic tissue homogenizer. Subsequently, the homogenized samples were subjected to freeze–thaw cycles to disrupt the cells further. The samples were centrifuged at 5000 rpm, and the supernatant was collected and stored at −80 °C for subsequent assays [57].

#### 4.6.4. Testis Histopathology Analysis

One testis was immersed in 10% formaldehyde on the day of sacrifice for histological evaluation. Five-micrometer-thick paraffin sections were cut and sent to Rapid Science Co., Ltd. (Brooklyn, NY, USA), for H&E staining.

#### 4.6.5. Malondialdehyde and Nitric Oxide Assay

Malondialdehyde (MDA) levels were used to evaluate lipid peroxidation according to previous study [51]. Plasma, sperm, and testis homogenate were mixed with the reactive solution (15% [*w*/*v*] trichloroacetic acid in 0.25 n-HCl and 0.375% (*w*/*v*) thiobarbituric acid in 0.25 n-HCl) and placed in a 100 °C water bath for 15 min. After cooling, the mixture was added to 300 μL n-butanol and centrifuged at 1500× *g* for 10 min. The absorbance of the clear supernatant was measured at 532 nm.

Nitric oxide (NO) production was assessed by measuring the stable end-product nitrite using Griess reagent [58]. The plasma, sperm, and testis tissue homogenates from each group (100 μL) were mixed with 100 μL of Griess reagent. The mixtures were then incubated at room temperature for 10 min. After incubation, absorbance was measured at 540 nm using a microplate spectrophotometer.

#### 4.6.6. Pro-Inflammatory Cytokines, Kisspeptin, and Reproductive Hormones Assay

TNF-α, IL-1β, and IL-6 levels in the plasma and testes were detected using enzyme-linked immunosorbent assay. Kisspeptin, GnRH, LH, FSH, and testosterone levels were measured in the plasma using ELISA kits. Additionally, the expression of 5-HT and 5-Htr1a in brain tissue was determined using ELISA kits. The analysis was performed in accordance with the manufacturer’s instructions.

### 4.7. Statistical Analysis

All data are expressed as mean ± standard deviation (SD) and were analyzed using one-way analysis of variance (one-way ANOVA) followed by Tukey multiple comparison tests. Differences were considered statistically significant at *p* < 0.05. Statistical analysis was performed using Statistical Product and Service Solutions v22.0 and GraphPad Prism v9 software.

## 5. Conclusions

In cell experiments, polystyrene microplastics (PS-MPs) have been shown to increase reactive oxygen species (ROS) levels and exhibit cytotoxic effects. Treatment with *Lactobacillus brevis* GKJOY postbiotics reduced the ROS levels in the Leydig cell line (LC540) and protected against PS-MP-induced oxidative stress. In animal experiments, *L. brevis* GKJOY effectively mitigated reproductive damage in male rats. *L. brevis* GKJOY exhibited antioxidant and anti-inflammatory properties. Additionally, *L. brevis* GKJOY supports reproductive health by improving hormone levels and sperm quality. Thus, *L. brevis* GKJOY offers a multifaceted approach to health by reducing oxidative stress and inflammation, improving hormone levels, and enhancing sperm quality, ultimately leading to improved reproductive health.

## Figures and Tables

**Table 1:** Organ weight of male rats after 8 weeks of treatments.

Organs (% of Body Weight)	Control	PS-MPs	GK1X	GK2X	GK4X	GABA
Liver	3.69 ± 0.32	3.84 ± 0.39	3.62 ± 0.37	3.73 ± 0.38	3.97 ± 0.50	3.82 ± 0.53
Spleen	0.14 ± 0.02	0.16 ± 0.03	0.15 ± 0.01	0.15 ± 0.01	0.16 ± 0.02	0.17 ± 0.03
Kidney	0.87 ± 0.08	0.84 ± 0.05	0.91 ± 0.06	0.76 ± 0.30	0.84 ± 0.11	0.88 ± 0.04
Abdominal adipose	1.61 ± 0.44	1.38 ± 0.27	1.61 ± 0.26	1.20 ± 0.32	1.39 ± 0.27	1.44 ± 0.32
Testis	0.71 ± 0.08	0.68 ± 0.07	0.76 ± 0.05	0.74 ± 0.07	0.70 ± 0.09	0.64 ± 0.06
Epididymal adipose	1.33 ± 0.26	1.09 ± 0.33	1.36 ± 0.27	1.11 ± 0.50	1.44 ± 0.14	1.34 ± 0.33

Data are shown as the mean ± SD (*n* = 6). There is no significance difference (*p* > 0.05) between groups as analyzed by the Tukey test. GABA, γ-aminobutyric acid; GK, *L. brevis* GKJOY; PS-MPs, polystyrene microplastics.

**Table 2:** The thickness of the epithelium and area of seminiferous lumen tubules in the testis of rats.

Parameters	Control	PS-MPs	GK1X	GK2X	GK4X	GABA
Thickness of the epithelium (μm)	114.00 ± 7.77 ^c^	62.19 ± 14.08 ^a^	71.77 ± 10.45 ^a^	107.65 ± 10.32 ^c^	104.93 ± 5.76 ^bc^	91.05 ± 4.41 ^b^
Area of seminiferous lumen/tubules (%)	3.28 ± 0.45 ^a^	31.57 ± 2.69 ^e^	23.63 ± 1.54 ^d^	10.85 ± 1.42 ^c^	7.73 ± 1.02 ^b^	12.89 ± 1.48 ^c^

Data are shown as the mean ± SD (*n* = 6). Values in a row with different superscript letters (a–e) are significantly different (*p* < 0.05) as analyzed by the Tukey test. GABA, γ-aminobutyric acid; GK, *L. brevis* GKJOY; PS-MPs, polystyrene microplastics.

## Data Availability

The original contributions presented in this study are included in the article. Further inquiries can be directed to the corresponding author.

## Abbreviations

The following abbreviations are used in this manuscript:

5-HT	5-hydroxytryptamine
5-Htr1a	5-hydroxytryptamine receptor 1A
FSH	Follicle-stimulating hormone
GABA	γ-aminobutyric acid
GK	*Lactobacillus brevis* GKJOY powder
GK1X	A low dose of *L. brevis* GKJOY (50 mg/kg)
GK2X	A medium dose of *L. brevis* GKJOY (100 mg/kg)
GK4X	A high dose of *L. brevis* GKJOY (200 mg/kg)
GnRH	Gonadotropin-releasing hormone
GPx	Glutathione peroxidase
IL	Interleukin
LH	Luteinizing hormone
MDA	Malondialdehyde
NO	Nitric oxide
PGK	Postbiotic metabolites of *Lactobacillus brevis* GKJOY
PS-MPs	Polystyrene microplastics
ROS	Reactive oxygen species
TNF	Tumor necrosis factor
